# Biologic therapy in geriatric psoriasis: 6-month real-world data on PASI, inflammatory indices, and hepatitis B/tuberculosis safety

**DOI:** 10.17305/bb.2026.13927

**Published:** 2026-03-04

**Authors:** Esranur Ünal, Beste Pakırdaşı, Selma Emre, Muhammed Burak Yücel, Burak Celik, Esma Nur Özbek, Nurşah Gedikli, Atıl Avcı, Ragıp Ertaş, Orhan Yıldız

**Affiliations:** 1 Department of Dermatology, Ufuk University, Faculty of Medicine, Ankara, Türkiye; 2 Department of Dermatology, University of Health Sciences, Kayseri City Education and Research Hospital, Kayseri, Türkiye; 3 Department of Internal Medicine, John H. Stroger, Jr. Hospital of Cook County, Chicago, IL, USA; 4 Department of Infectious Diseases and Clinical Microbiology, Erciyes University, Faculty of Medicine, Kayseri, Türkiye

**Keywords:** Geriatric psoriasis, biologic therapy, PASI, systemic inflammation, systemic immune-inflammation index, QuantiFERON-TB, hepatitis B

## Abstract

Real-world evidence regarding biologic therapy in geriatric psoriasis is limited, particularly concerning systemic inflammatory burden and infection-related safety. This study evaluates the clinical efficacy of biologic therapy and its impact on systemic inflammatory indices while emphasizing safety related to hepatitis B virus (HBV) serology and tuberculosis screening. We conducted a retrospective analysis of eighty biologic-naïve patients aged 65 years and older with plaque psoriasis undergoing biologic therapy. Patients were categorized by biologic class: tumor necrosis factor-α (TNF-α) inhibitors, interleukin-17 inhibitors, an interleukin-12/23 inhibitor, and interleukin-23 inhibitors. The primary outcomes included changes in Psoriasis Area and Severity Index (PASI) scores and blood count-derived inflammatory indices over time (baseline and 6 months). Secondary outcomes encompassed changes in HBV serologic status and QuantiFERON-TB (QFT) results. Data analysis utilized longitudinal mixed-effects models for repeated measures. Blood count-derived inflammatory indices, such as neutrophil-to-lymphocyte ratio (NLR), platelet-to-lymphocyte ratio (PLR), systemic immune-inflammation index (SII), and systemic inflammation response index (SIRI), were assessed at baseline and 6 months, alongside HBV serology and QFT results. PASI scores demonstrated significant improvement over time (*P <* 0.001), with no notable differences among biologic classes after adjusting for baseline covariates. Significant time effects were observed for all inflammatory indices (all *P <* 0.001), with significant group × time interactions noted for SII and NLR (both *P <* 0.05). Variability in HBV serologic markers and QFT results was observed during follow-up; however, no cases of active tuberculosis or clinically overt hepatitis were identified. In conclusion, biologic therapy led to substantial clinical improvement in geriatric psoriasis, accompanied by reductions in systemic inflammatory indices over a 6-month period, without evidence of clinically overt hepatitis or active tuberculosis during follow-up.

## Introduction

Psoriasis is a chronic, immune-mediated inflammatory skin disease characterized by various clinical phenotypes. Its pathogenesis is multifactorial, involving genetic predisposition, epigenetic regulation, immune dysregulation, alterations in the microbiome, and environmental triggers [1]. With an estimated global prevalence of approximately 2%, psoriasis typically manifests as erythematous, scaly plaques, significantly impacting both quality of life and healthcare utilization [2].

The aberrant activation of innate and adaptive immune pathways is fundamental to the initiation and persistence of psoriatic inflammation. Key cytokine pathways, such as tumor necrosis factor (TNF)-α and the IL-23/IL-17 axis, are upregulated in lesional skin and systemic circulation, making them major therapeutic targets [3, 4]. Notably, psoriasis extends beyond skin involvement; chronic inflammation may lead to systemic immune activation and the emergence of psoriasis-associated comorbidities, a phenomenon referred to as the “psoriatic march” [5, 6]. Psoriasis is associated with psoriatic arthritis, metabolic and cardiovascular disorders, and depression [6, 7]. In geriatric patients (aged ≥65 years), the presence of multiple comorbidities, polypharmacy, and age-related pharmacokinetic and pharmacodynamic changes complicate treatment selection and heighten the importance of evaluating both efficacy and safety [1, 8]. The challenges posed by multiple comorbidities, polypharmacy, and heightened risks of infections and malignancies underscore the complexity of treatment decision-making in elderly patients, while specific treatment guidelines for this demographic remain limited, emphasizing the need for real-world data [9]. Recent studies in elderly psoriasis populations have revealed a significant comorbidity burden and suggested that biologic therapies can remain effective and safe despite the presence of multimorbidity [10].

Despite advancements in understanding inflammatory pathways, psoriasis currently lacks reliable laboratory biomarkers that reflect disease activity and treatment response. Consequently, disease monitoring predominantly relies on clinical scoring systems, such as the Psoriasis Area and Severity Index (PASI) [11]. While real-world studies often assess treatment effectiveness through drug survival metrics, clinical efficacy measures like PASI continue to be crucial for evaluating biological responses. In this context, systemic inflammatory indices derived from routine blood counts—such as the neutrophil-to-lymphocyte ratio (NLR), platelet-to-lymphocyte ratio (PLR), systemic immune-inflammation index (SII), and systemic inflammation response index (SIRI)—have been proposed as surrogate markers of systemic inflammation [6, 11, 12]. Although biologic therapies may lower these indices, their relationship with clinical severity and their utility in guiding treatment decisions remain inadequately defined [4, 6].

Biologic therapies targeting TNF-α and the IL-23/IL-17 axis have markedly improved outcomes in moderate-to-severe psoriasis [1, 13]. However, evidence regarding their efficacy and safety in geriatric populations is limited due to the frequent underrepresentation of these patients in clinical trials and the scarcity of real-world safety data [14, 15]. In addition to efficacy, the safety profile of biologics in elderly patients requires thorough evaluation, particularly concerning the potential for infection-related complications. Immunomodulatory therapies may elevate the risk of reactivation of hepatitis B virus (HBV) and latent tuberculosis infection (LTBI), influenced by host factors and the specific biologic agent used [16, 17]. Therefore, appropriate screening prior to treatment initiation is critical to mitigate reactivation risks while avoiding unnecessary prophylactic interventions in uninfected individuals [17].

The primary aim of this study was to evaluate the effects of biologic agents on disease severity, as assessed by PASI, and on systemic inflammation in biologic-naïve geriatric patients with psoriasis across various biologic classes and agents in a real-world tertiary care setting, utilizing blood count-derived inflammatory indices. The secondary aim was to assess the short-term safety profile of biologic therapy, including adverse events and infection-related findings, with particular emphasis on dynamic changes in HBV status and LTBI screening results assessed by the QuantiFERON-TB (QFT) test.

## Materials and methods

**Figure 1.**
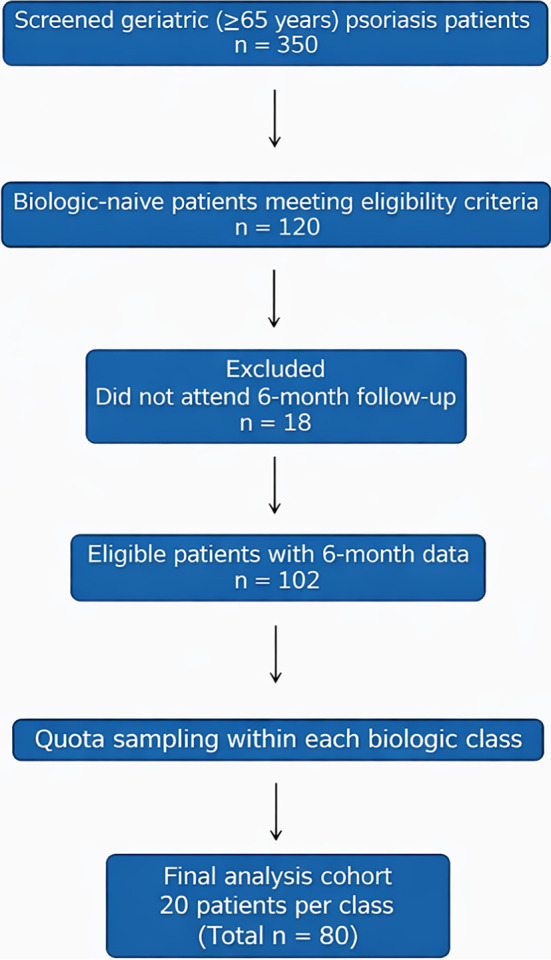
**Flow diagram of patient selection.** A total of 350 geriatric patients (≥65 years) with psoriasis were screened. Of these, 120 biologic-naïve patients met the eligibility criteria. Eighteen patients who failed to attend the 6-month follow-up visit were excluded. From the remaining eligible cohort (*n* ═ 102), 20 patients from each biologic class were selected using quota sampling to establish balanced treatment groups for the final analysis (total *n* ═ 80).

This retrospective study included 80 patients aged ≥65 years with plaque psoriasis, diagnosed clinically and/or histopathologically, who received biologic therapy between March 2014 and March 2024. During the study period, 350 geriatric psoriasis patients were screened, of whom 120 were biologic-naïve. Patients without at least 6 months of follow-up were excluded (*n* ═ 18). Quota sampling was applied to obtain balanced biologic groups, resulting in 20 patients per class (total *n* ═ 80). A flow diagram summarizing patient selection is presented in Figure 1.

Biologic agents were classified into four groups based on their mechanisms of action: TNF-α inhibitors (infliximab, adalimumab, etanercept), IL-17 inhibitors (secukinumab, ixekizumab), an IL-12/23 inhibitor (ustekinumab), and IL-23 inhibitors (guselkumab, risankizumab). Patients with active malignancies, severe infections, prior biologic therapy, inability to adhere to regular follow-up, or missing paired baseline and 6-month data were excluded.

All patients had previously received at least one conventional systemic therapy and were escalated to biologic treatment due to inadequate response, loss of efficacy, or treatment-related adverse events. Patients with PASI scores <10 were included when biologic therapy was deemed clinically appropriate due to special area involvement or intolerance to conventional systemic therapies. Demographic and clinical data were obtained from electronic medical records. Recorded variables included age, sex, body mass index (BMI), disease duration, presence of psoriatic arthritis and nail involvement, comorbidities, and the type of biologic agent used. Disease severity was classified according to PASI scores, with scores >10 indicating moderate-to-severe disease and scores ≤10 classified as mild disease [18].

Clinical and laboratory evaluations were conducted at baseline and at 6 months. Clinical response was primarily assessed through changes in PASI scores over time. Additionally, PASI 75, PASI 90, and PASI 100 response rates (indicating 75%, 90%, and 100% reductions in PASI, respectively) were recorded as descriptive measures of clinical efficacy. Systemic inflammatory indices, including NLR, PLR, SII, and SIRI, were calculated at both time points. Clinical outcomes and inflammatory indices were also evaluated across biologic classes and individual agents.

NLR and PLR were calculated as the ratios of neutrophil and platelet counts to lymphocyte count, respectively. SII was calculated as (platelet count × neutrophil count) / lymphocyte count, and SIRI as (neutrophil count × monocyte count) / lymphocyte count, based on complete blood count parameters [19, 20].

Evaluated parameters included liver function tests [aspartate aminotransferase (AST), alanine aminotransferase (ALT), alkaline phosphatase (ALP), and gamma-glutamyl transferase (GGT)], renal function tests (urea and creatinine), electrolyte levels (sodium, potassium, magnesium, and calcium), lipid profile [high-density lipoprotein (HDL), low-density lipoprotein (LDL), and triglycerides], erythrocyte sedimentation rate (ESR), and C-reactive protein (CRP). Infectious disease screening encompassed hepatitis serology, which included the following tests: hepatitis B surface antigen (HBsAg), antibody to hepatitis B surface antigen (anti-HBs), total antibody to hepatitis B core antigen (total anti-HBc), antibody to hepatitis C virus (anti-HCV), and antibody to human immunodeficiency virus (anti-HIV). Additionally, QFT Gold testing was performed.

HBV DNA levels were measured using a quantitative real-time polymerase chain reaction assay and reported as IU/mL. The lower limit of detection of the assay was 31 IU/mL, with values below this limit recorded as <31 IU/mL. HBV reactivation was defined as a ≥2 log increase in HBV DNA levels in HBsAg-positive patients with detectable baseline HBV DNA or the reappearance of HBV DNA exceeding 100 IU/mL in those with undetectable baseline HBV DNA. In HBsAg-negative/anti-HBc–positive patients, HBV reactivation was defined as the reappearance of HBsAg or detection of previously undetectable HBV DNA exceeding 100 IU/mL [21]. All patients with evidence of inactive or resolved HBV infection (HBsAg-positive and/or anti-HBc–positive) received antiviral prophylaxis during biologic therapy. HBV-seronegative patients were advised to receive hepatitis B vaccination as part of routine clinical care, and anti-HBs levels were reassessed during follow-up to document seroconversion. HBV serologic markers and HBV DNA levels were monitored approximately every 3 months (±2 weeks) during biologic therapy as part of routine clinical safety monitoring.

LTBI screening was performed using the QFT Gold assay (QIAGEN, Hilden, Germany) and repeated every 6 months during biologic therapy. Patients with a positive baseline QFT received isoniazid prophylaxis prior to initiation of biologic therapy. If QFT results became positive during follow-up, isoniazid prophylaxis was initiated. Serious infections and malignancies during follow-up were identified through reviews of electronic medical records, including both hospitalization and outpatient records. These evaluations were conducted as part of routine clinical safety monitoring during biologic therapy.

### Ethical statement

Ethical approval for this study was obtained from the institutional ethics committee on April 18, 2024 (decision number: 63).

### Statistical analysis

Statistical analyses were conducted using IBM SPSS Statistics, the Statistical Package for the Social Sciences (Version 26.0; IBM Corp., Armonk, NY, USA). Continuous variables were presented as mean ± standard deviation or median (interquartile range, IQR), while categorical variables were expressed as frequencies and percentages. Data distribution was assessed using the Shapiro–Wilk test. Continuous variables were compared with the independent samples *t*-test or Mann–Whitney *U* test for two independent groups, and the Kruskal–Wallis test for comparisons involving more than two groups. For dependent samples, the paired-samples *t*-test or Wilcoxon signed-rank test was employed. Categorical variables were analyzed using Pearson’s chi-square test, Fisher’s exact test, or the Fisher–Freeman–Halton test as appropriate.

Longitudinal changes in clinical outcomes (PASI) and systemic inflammatory indices (SII, NLR, PLR, and SIRI) were analyzed using linear mixed-effects models (LMMs) to account for repeated measurements within individuals [22]. Separate models were constructed for PASI and each inflammatory index. Fixed effects included time (baseline vs. month 6), biologic treatment group, and their interaction. Models were adjusted for potential confounders, including age, sex, disease duration, psoriatic arthritis status, comorbidities, baseline CRP, and baseline PASI or baseline inflammatory index, as appropriate. Prior treatment exposure was not included as a covariate. A compound symmetry covariance structure was specified, and parameters were estimated using restricted maximum likelihood (REML). Estimated marginal means were calculated, and pairwise comparisons were performed with Bonferroni correction. The Benjamini–Hochberg false discovery rate procedure was applied to the p-values of the fixed effects within each effect category to control for multiplicity across the four inflammatory indices. For other secondary and exploratory analyses, p-values were interpreted descriptively without formal adjustment for multiple comparisons. A two-sided p-value <0.05 was considered statistically significant.

## Results

### Demographic and clinical characteristics

A total of 80 geriatric patients with psoriasis receiving biologic therapy were included in this study. The median age was 66 years (IQR: 65–69), with 46 patients (57.5%) being male. The most prevalent comorbidities included diabetes mellitus, hypertension, hyperlipidemia, cardiovascular disease, obesity, thyroid disease, and chronic obstructive pulmonary disease (COPD). Baseline characteristics are summarized in Table 1.

**Table 1:** Baseline demographic and clinical characteristics of geriatric psoriasis patients by biologic class

**Variable**	**TNF-α inhibitors (*n* ═ 20)**	**IL-12/23 inhibitors (*n* ═ 20)**	**IL-17 inhibitors (*n* ═ 20)**	**IL-23 inhibitors (*n* ═ 20)**	**Total** **(*n* ═ 80)**	***P* value**
**Age,** years, median (IQR)	66 (65–66.75)	66 (65–69.75)	65.5 (65–69)	66 (65–71)	66 (65–69)	0.694^x^
**Sex**, *n* (%)
● Male	9 (45%)	11 (55%)	15 (75%)	11 (55%)	46 (57.5%)	0.274^y^
● Female	11 (55%)	9 (45%)	5 (25%)	9 (45%)	34 (42.5%)
**Disease duration**, months, median (IQR)	222 (126--345)	186 (120--279)	168 (111--300)	144 (108--258)	180 (120--296)	0.400^x^
**Baseline PASI score,** median (IQR)	6.5 (5--10)	9.5 (7.85--12.45)	10.9 (5.75--16.5)	9.8 (6.25--14.95)	9.8 (6--13.75)	0.149^x^
**Disease severity,** *n* (%)
● PASI ≤ 10	16 (80%)	11 (55%)	9 (45%)	11 (55%)	47 (59%)	0.138^y^
● PASI > 10	4 (20%)	9 (45%)	11 (55%)	9 (45%)	33 (41%)
**Psoriatic arthritis**, *n* (%)	6 (30%)	4 (20%)	6 (30%)	4 (20%)	20 (25%)	0.785^y^
**Nail involvement**, *n* (%)	20 (100%)	19 (95%)	17 (85%)	20 (100%)	76 (95%)	0.185^z^

Data are presented as median (IQR) or *n* (%). ^*χ*^Kruskal–Wallis test; ^y^χ^2^ test; ^z^Fisher–Freeman–Halton exact test. Abbreviations: TNF-α: Tumor necrosis factor alpha; IL: Interleukin; IQR: Interquartile range; PASI: Psoriasis Area and Severity Index; χ^2^: Chi-square test.

### Biologic therapies

**Figure 2.**
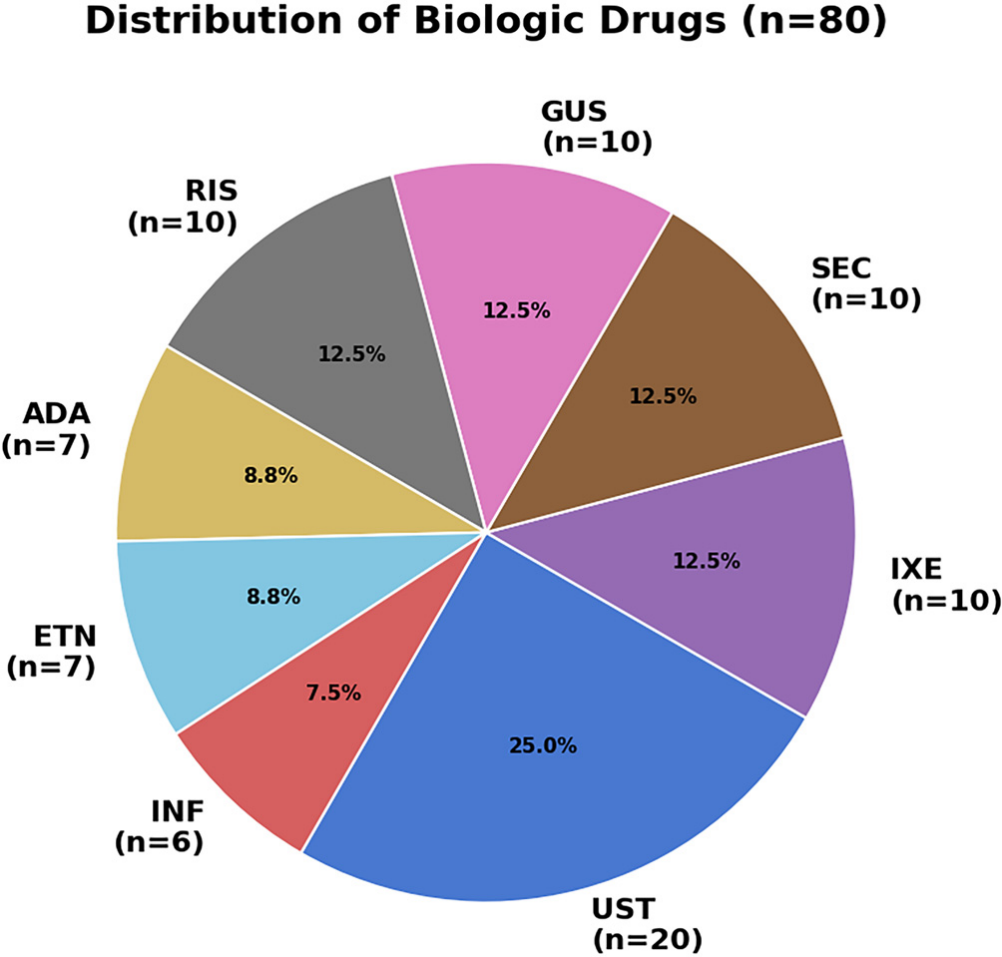
**Distribution of individual biologic agents in the study cohort (*n* ═ 80).** Values are presented as *n* (% of the total cohort) for each agent. The cohort was balanced by biologic class (*n* ═ 20 per class): TNF-α inhibitors (adalimumab, etanercept, infliximab), IL-17 inhibitors (secukinumab, ixekizumab), the IL-12/23 inhibitor (ustekinumab), and IL-23 inhibitors (guselkumab, risankizumab). Abbreviations: ADA: Adalimumab; ETN: Etanercept; INF: Infliximab; SEC: Secukinumab; IXE: Ixekizumab; UST: Ustekinumab; GUS: Guselkumab; RIS: Risankizumab; TNF-α: Tumor necrosis factor alpha; IL: Interleukin.

Biologic therapies were classified into four groups (*n* ═ 20 each): TNF-α inhibitors (adalimumab, etanercept, infliximab), IL-17 inhibitors (secukinumab, ixekizumab), IL-12/23 inhibitors (ustekinumab), and IL-23 inhibitors (guselkumab, risankizumab). All patients continued their initial biologic therapy throughout the 6-month follow-up period. The distribution of biologic therapies is illustrated in Figure 2.

### Clinical efficacy and PASI responses at 6 months

The median PASI decreased significantly from 9.8 (IQR: 6–13.75) at baseline to 1 (IQR: 0–2) at 6 months (*P* < 0.001).

In the LMM incorporating time, biologic class, and a time-by-class interaction term, a significant effect of time on PASI scores was observed (*P <* 0.001), as detailed in Table 2. The overall effect of biologic class was not statistically significant (*P ═* 0.514), and the time-by-class interaction was also non-significant (*P ═* 0.245).

**Table 2:** Linear mixed-effects model analysis of PASI changes over time

**Variable**	**Estimate**	**SE**	**95% CI**	***P∘***	*P* ^*^
Time	—	—	—	**<0.001**	
Time (Baseline)	10.060	1.446	7.179; 12.941		**<0.001**
Treatment group	—	—	—	0.514	
Time × Treatment group	—	—	—	0.245	
Sex (male)	1.581	0.967	--0.351; 3.513		0.107
Age	--0.034	0.129	--0.292; 0.223		0.791
Disease duration	--0.005	0.002	--0.011; 0.001		0.089
CRP (baseline)	--0.083	0.077	--0.237; 0.072		0.291
Psoriatic arthritis	--0.742	0.941	--2.621; 1.135		0.432
COPD	0.067	1.027	--1.983; 2.118		0.948
CAD	--0.902	0.927	--2.753; 0.95		0.335
DM	0.843	0.82	--0.794; 2.48		0.308
Hypertension	--1.404	0.877	--3.155; 0.348		0.114
SII (baseline)	0.003	0.001	0.0002; 0.0061		**0.036**

LMM was employed for analysis, utilizing reference categories of Time (6 months) and Treatment Group (IL-23 inhibitor). *P∘* (Type III F-test): Indicates the overall statistical significance of each fixed effect. P* (Fixed Effect Estimate): Represents the significance of the specific parameter estimate. Abbreviations: PASI: Psoriasis Area and Severity Index; LMM: Linear mixed model; SE: Standard error; CI: Confidence interval; CRP: C-reactive protein; SII: Systemic immune-inflammation index; COPD: Chronic obstructive pulmonary disease; CAD: Coronary artery disease; DM: Diabetes mellitus; IL: Interleukin; TNF-α: Tumor necrosis factor alpha.

Six-month PASI response thresholds (PASI 75, PASI 90, and PASI 100) according to biologic class are presented in Table 3.

### Factors associated with PASI outcomes

Among the covariates included in the model, higher baseline SII levels were independently associated with increased PASI scores (β ═ 0.003, 95% CI: 0.0002–0.0061; *P ═* 0.036).

**Table 3:** Response rates for PASI75, PASI90, and PASI100 by biologic class at 6 months

**Group**	***n***	**PASI 75**	**PASI 90**	**PASI 100**
TNF-α inhibitors	20	17 (85%; 62.1–96.8)	13 (65%; 40.8–84.6)	13 (65%; 40.8–84.6)
IL-12/23 inhibitors	20	13 (65%; 40.8–84.6)	5 (25%; 8.7–49.1)	5 (25%; 8.7–49.1)
IL-17 inhibitors	20	19 (95%; 75.1–99.9)	15 (75%; 50.9–91.3)	10 (50%; 27.2–72.8)
IL-23 inhibitors	20	15 (75%; 50.9–91.3)	10 (50%; 27.2–72.8)	7 (35%; 15.4–59.2)
Total	80	64 (80%; 69.6–88.1)	43 (53.8%; 42.2–65)	35 (43.8%; 32.7–55.3)

Data are presented as number (%). The PASI is utilized in this analysis. In specific treatment groups, the PASI90 and PASI100 values were identical, as no patients achieved responses within the 90% to 99% improvement range. Exact 95% confidence intervals were determined using the Clopper–Pearson method. Abbreviations: PASI: Psoriasis Area and Severity Index; PASI75: ≥75% reduction in PASI; PASI90: ≥90% reduction in PASI; PASI100: 100% reduction in PASI (complete clearance); TNF-α: Tumor necrosis factor alpha; IL: Interleukin; CI: Confidence interval.

### Systemic inflammatory indices

In the LMMs, no significant main effect of biologic class was observed for SII or NLR (*P ═* 0.652 and *P ═* 0.621, respectively), while a significant main effect of time was evident for both indices (*P <* 0.001 for both). Furthermore, significant biologic class × time interactions were identified for SII (*P ═* 0.019) and NLR (*P*= 0.012), as presented in Table 4.

**Table 4:** Analysis of SII and NLR using linear mixed-effects models

	**SII**				
**Variable**	**Estimate**	**SE**	**95% CI**	***P∘***	*P* ^*^
Time	—	—	—	**<0.001**	
Time (baseline)	172.54	43.42	86.05; 259.04		**<0.001**
Treatment group (overall)	—	—	—	0.652	
Time × Treatment	—	—	—	**0.019^a^**	
Time (baseline)*TNF inh.	--180.65	61.42	--302.97; --58,32		**0.004**
Time (baseline)*IL12-23 inh.	--23,88	61.42	--146.20; 98.45		0.699
Time (baseline)*IL17 inh.	--39.09	61.42	--161.42; 83.23		0.526
PASI (baseline)	7.91	5.18	--2.42; 18.24		0.131
Disease duration	--0.45	0.24	--0.93; 0.03		0.067
Sex (Male)	--82.74	79.27	--214.03; 75.54		0.300
Age	3.55	10.51	--17.45; 24.55		0.737
Psoriatic Arthritis	15.70	76.18	--136.41; 167.82		0.837
COPD	88.59	83.04	--77.21; 254.38		0.290
CAD	--34.83	76.04	--186.65; 116.97		0.648
DM	--61.40	67.14	--195.45; 72.65		0.364
Hypertension	50.59	72.86	--94.88; 196.06		0.490
CRP (baseline)	3.48	6.37	--9.23; 16.19		0.587
	**NLR**				
**Variable**	**Estimate**	**SE**	**95% CI**	***P∘***	*P* ^*^
Time	—	—	—	**<0.001**	
Time (baseline)	0.455	0.157	0.141; 0.769		**0.005**
Treatment group (overall)	—	—	—	0.621	
Time × Treatment	—	—	—	**0.012^b^**	
Time (baseline)*TNF inh.	--0.643	0.222	--1.087; --0.199		**0.005**
Time (baseline)*IL12-23 inh.	--0.003	0.222	--0.447; 0.440		0.989
Time (baseline)*IL17 inh.	--0.064	0.222	--0.508; 0.379		0.774
PASI (baseline)	0.032	0.017	--0.002; 0.067		0.069
Disease duration	--0.001	0.001	--0.0030; 0.0002		0.094
Sex (Male)	0.177	0.267	--0.356; 0.712		0.509
Age	0.012	0.035	--0.058; 0.083		0.728
Psoriatic Arthritis	0.184	0.257	--0.329; 0.698		0.476
COPD	0.152	0.280	--0.407; 0.712		0.589
CAD	--0.346	0.256	--0.859; 0.165		0.181
DM	--0.378	0.226	--0.831; 0.074		0.100
Hypertension	0.284	0.246	--0.206; 0.775		0.252
CRP (baseline)	--0.002	0.021	--0.045; 0.041		0.927

LMM was employed for the analysis, with reference categories set for Time (6 months) and Treatment Group (IL-23 inhibitor). *P∘* (Type III F-test): Indicates the overall statistical significance of each fixed effect. P* (Fixed Effect Estimate): Represents the significance of the specific parameter estimate. *P* values were computed using exact raw data, and the displayed values are rounded to three decimal places. ^a^Raw *P* value = 0.019069; ^b^Raw *P* value = 0.011837. Abbreviations: SII: Systemic immune-inflammation index; NLR: Neutrophil-to-lymphocyte ratio; LMM: Linear mixed model; SE: Standard error; CI: Confidence interval; PASI: Psoriasis Area and Severity Index; CRP: C-reactive protein; COPD: Chronic obstructive pulmonary disease; CAD: Coronary artery disease; DM: Diabetes mellitus; TNF-α: Tumor necrosis factor alpha; IL: Interleukin.

For PLR and SIRI, a significant effect of time was also observed (*P <* 0.001 for both), while neither the main effect of biologic class nor the biologic class × time interaction reached statistical significance (all *P >* 0.05). These results are shown in Table 5.

**Table 5:** Analysis of PLR and SIRI using linear mixed-effects model

	**PLR**				
**Variable**	**Estimate**	**SE**	**95% CI**	***P∘***	*P* ^*^
Time	—	—	—	**<0.001**	
Time (baseline)	20.09	6.50	7.14; 33.03		**0.003**
Treatment group (overall)	—	—	—	0.936	
Time × Treatment	—	—	—	0.328	
PASI (baseline)	1.01	0.77	--0.53; 2.56		0.195
Disease duration	--0.06	0.04	--0.13; 0.01		0.106
Sex (Male)	--13.79	11.85	--37.45; 9.88		0.249
Age	0.70	1.57	--2.44; 3.84		0.445
Psoriatic Arthritis	0.65	11.39	--22.09; 23.39		0.954
COPD	12.76	12.42	--12.03; 37.55		0.308
CAD	--0.55	11.37	--23.25; 22.15		0.962
DM	--17.97	10.04	--38.01; 2.08		0.078
Hypertension	6.34	10.89	--15.41; 28.09		0.562
CRP (baseline)	0.17	0.95	--1.73; 2.07		0.859
	**SIRI**				
**Variable**	**Estimate**	**SE**	**95% CI**	***P∘***	*P* ^*^
Time	—	—	—	**<0.001**	
Time (baseline)	0.64	0.13	0.38; 0.91		**<0.001**
Treatment group (overall)	—	—	—	0.672	
Time × Treatment	—	—	—	0.258	
PASI (baseline)	0.013	0.014	--0.014; 0.041		0.338
Disease duration	--0.001	0.001	--0.0022; 0.0003		0.159
Sex (Male)	0.183	0.211	--0.239; 0.606		0.390
Age	0.054	0.028	--0.001; 0.110		0.057
Psoriatic arthritis	0.084	0.204	--0.322; 0.491		0.680
COPD	0.199	0.221	--0.244; 0.641		0.374
CAD	--0.077	0.203	--0.483; 0.327		0.703
DM	--0.016	0.179	--0.374; 0.341		0.926
Hypertension	0.050	0.194	--0.338; 0.439		0.796
CRP (baseline)	0.008	0.017	--0.025; 0.042		0.635

LMM was employed for analysis, utilizing the following reference categories: Time (6 months) and Treatment Group (IL-23 inhibitor). *P∘* (Type III F-test): Indicates the overall statistical significance of each fixed effect. P* (Fixed Effect Estimate): Represents the significance of the specific parameter estimate. Abbreviations: PLR: Platelet-to-lymphocyte ratio; SIRI: Systemic inflammation response index; LMM: Linear mixed model; SE: Standard error; CI: Confidence interval; PASI: Psoriasis Area and Severity Index; CRP: C-reactive protein; COPD: Chronic obstructive pulmonary disease; CAD: Coronary artery disease; DM: Diabetes mellitus; IL: Interleukin.

After adjustment for multiple comparisons using the Benjamini–Hochberg false discovery rate procedure, the main effect of time remained significant for all four indices (adjusted *P <* 0.001 for all). The biologic class × time interaction also remained significant for SII (adjusted *P ═* 0.038) and NLR (adjusted *P ═* 0.047). Compared to the IL-23 inhibitor group (reference category), the TNF-α inhibitor group exhibited a significantly smaller reduction over time in both SII and NLR.

### Laboratory parameters

No significant changes were observed in creatinine, urea, AST, ALT, calcium, sodium, potassium, magnesium, LDL, HDL, triglycerides, or CRP from baseline to 6 months (all *P* > 0.05). ESR decreased significantly (median change ═ −1.5; 95% CI: --3 to --0.5, *P* ═ 0.01), while ALP (median change = 5.5; 95% CI: 3 – 8.5, *P <* 0.001) and GGT (median change = 2.5; 95% CI: 1 – 4.5, *P* ═ 0.001) increased significantly.

### Infectious safety outcomes

HBV reactivation occurred in four patients (5.0%, 95% CI: 1.4–12.3) during the 6-month follow-up period, all of whom were receiving antiviral prophylaxis at baseline. Three patients had serologic evidence of natural immunity, while one had a chronic HBV infection at baseline. HBV reactivation was observed in patients receiving infliximab (*n* ═ 1), etanercept (*n* ═ 2), and guselkumab (*n* ═ 1). Notably, ALT and AST levels remained within normal ranges in all cases, and no clinically evident hepatitis was observed. HBV serologic and virologic marker profiles in patients with HBV reactivation are shown in Table 6; overall HBV clinical status during follow-up is summarized in Table 7.

**Table 6:** Changes in hepatitis B serologic markers and quantitative HBV DNA levels at baseline and six-month follow-up in patients with HBV reactivation

	**Baseline**					**6th month**				
**Patient**	**HbsAg**	**Anti-Hbs**	**Anti-Hbc total**	**HBV-DNA** **(IU/mL)**	**HbsAg**	**Anti-Hbs**	**Anti-Hbc total**	**HBV-DNA (IU/mL)**	**Biological agent**	**Antiviral prophylaxis**
Patient 1	+	--	+	<31	+	--	+	257	Etanercept	Entecavir
Patient 2	--	+	+	<31	--	--	+	179	Infliximab	Entecavir
Patient 3	--	+	+	<31	--	--	+	1292	Etanercept	Tenofovir
Patient 4	--	+	+	<31	+	--	+	241	Guselkumab	Entecavir

HBV DNA was quantified using a real-time PCR assay, with results expressed in international units per milliliter (IU/mL). The lower LOD was established at 31 IU/mL, and values falling below this threshold are reported as <31 IU/mL. Abbreviations: HBsAg: Hepatitis B surface antigen; anti-HBs: Antibody to hepatitis B surface antigen; anti-HBc: Antibody to hepatitis B core antigen; HBV: Hepatitis B virus; HBV DNA: Hepatitis B virus deoxyribonucleic acid; PCR: Polymerase chain reaction; LOD: Limit of detection.

**Table 7:** Overall hepatitis B serologic status at baseline and 6 months

**HBV status**	**Baseline** **(*n*)**	**6th month** **(*n*)**
**Seronegative**	28	8
**Vaccine immunity**	16	36
**Natural immunity**	32	29
**Chronic HBV infection**	4	5*
**Isolated anti-HBc positivity (HBV DNA detectable)**	0	2*
**Total**	80	80

Four patients experienced HBV reactivation during follow-up, as detailed in Table 6. Among those who were seronegative at baseline, 20 received hepatitis B vaccination and subsequently developed anti-HBs positivity. In contrast, among patients with natural immunity at baseline, three experienced HBV reactivation, while one patient with chronic HBV infection also exhibited reactivation during follow-up. Serologic category definitions: Seronegative: HBsAg (--), anti-HBs (--), anti-HBc (--); Vaccine-induced immunity: HBsAg (--), anti-HBs (+), anti-HBc (--); Natural immunity: HBsAg (--), anti-HBs (+), anti-HBc (+); Chronic HBV infection: HBsAg (+) regardless of antibody status; Isolated anti-HBc positivity (HBV DNA detectable): HBsAg (--), anti-HBs (--), anti-HBc (+) with detectable HBV DNA (≥31 IU/mL). Abbreviations: HBV: Hepatitis B virus; HBsAg: Hepatitis B surface antigen; anti-HBs: Antibody to hepatitis B surface antigen; anti-HBc: Antibody to hepatitis B core antigen; HBV DNA: Hepatitis B virus deoxyribonucleic acid.

Serologic screening for other viral infections revealed that anti-HCV positivity was present at both baseline and 6 months in two patients, whereas anti-HIV results remained negative in all patients throughout the follow-up period. Concerning LTBI, QFT results converted from negative to positive in 4 of 64 patients with baseline negative results (6.2%, 95% CI: 1.7–15.2), while 16 patients who were positive at baseline remained positive at 6 months. These conversions occurred in patients receiving risankizumab (*n* ═ 1), secukinumab (*n* ═ 2), and guselkumab (*n* ═ 1); however, this change did not reach statistical significance (*P >* 0.05). No cases of active tuberculosis were observed during the 6-month follow-up period.

No treatment discontinuations, serious infections, or malignancies were reported during the 6-month follow-up period.

## Discussion

Psoriasis is a chronic immune-mediated inflammatory disease characterized by cutaneous and systemic involvement, driven by dysregulated innate and adaptive immunity and key cytokine pathways, including TNF-α and the IL-23/IL-17 axis [23]. In geriatric patients, persistent inflammation contributes to comorbidity burden, while immunosenescence, polypharmacy, and multimorbidity complicate treatment decisions and safety monitoring [24–26]. In this real-world geriatric cohort, biologic therapy was associated with a substantial improvement in clinical disease activity over 6 months. PASI scores improved significantly over time without statistically significant differences among biologic classes. Additionally, significant reductions in blood count-derived systemic inflammatory indices were observed over time.

Previous studies suggest that the efficacy and safety of biologic therapies in geriatric psoriasis are comparable to those observed in younger populations [26]. Real-world evidence indicates that clinical responses in geriatric patients may initially be slower but improve with continued treatment and longer follow-up, particularly with IL-17 and IL-23 inhibitors [27, 28]. Consistent with these observations, PASI scores decreased significantly at 6 months in our cohort, supporting the clinical efficacy of biologic therapy in geriatric patients under real-world conditions. Notably, no statistically significant differences were detected between biologic classes, suggesting broadly comparable treatment responses across different biologic mechanisms in this population. These findings should be interpreted with caution given the relatively small sample size and the inherent heterogeneity within real-world geriatric cohorts, including variations in baseline disease characteristics, comorbidity burden, treatment history, and age-related pharmacodynamic factors. Nevertheless, longer-term follow-up is warranted to evaluate the durability of treatment responses.

Reliable biomarkers that accurately reflect disease activity and treatment response in psoriasis remain an unmet need. Blood count-derived indices are particularly attractive due to their cost-effectiveness, widespread availability, and ease of repeatability [6]. Real-world studies assessing the impact of biologic therapy on systemic inflammatory indices have generally reported reductions in SII and/or SIRI, although findings vary across biologic classes and patient cohorts [4, 23, 29–31]. For instance, decreases in SII and SIRI have been noted with inhibitors of TNF-α, IL-17, and IL-12/23 [4], while results for IL-23 inhibitors and specific indices have shown less consistency across reports [4, 29, 32].

NLR and PLR have been explored as accessible markers of systemic inflammation in psoriasis. However, the reported changes following biologic therapy remain heterogeneous. Some studies indicate significant reductions in both indices without clear distinctions among biologic agents [23], while others suggest class- and/or agent-specific patterns. For example, decreases in both NLR and PLR have been observed in patients receiving TNF-α inhibitors [30], with a notable reduction in PLR reported within this group [33]. Conversely, findings in adalimumab-treated patients revealed a significant reduction in NLR but not in PLR [34]. In contrast, increases in NLR and PLR have also been reported in cohorts treated with IL-17 and IL-23 inhibitors [35].

In our cohort, biologic therapy was associated with a reduction in systemic inflammatory burden among geriatric patients with psoriasis, as evidenced by decreases in SII, SIRI, NLR, and PLR over the six-month follow-up period. Significant improvements were observed for all indices, suggesting a general enhancement in systemic inflammation during treatment. Although overall differences between biologic classes were not statistically significant, the magnitude of change varied across treatment groups for SII and NLR, with patients receiving TNF-α inhibitors showing smaller reductions compared to those on IL-23 inhibitors.

The relatively smaller changes in the TNF-αinhibitor group may be attributed to age-related immune alterations and the diverse clinical characteristics of older patients, including a higher burden of comorbidities. In contrast, biologic agents targeting the IL-23/IL-17 pathway may more effectively modulate key inflammatory pathways involved in psoriasis pathogenesis, leading to greater improvements in systemic inflammatory markers. However, these differences should be interpreted cautiously due to the observational nature and limited sample size of the study.

Routine laboratory parameters, including renal function (urea and creatinine), aminotransferases (AST and ALT), electrolytes (sodium, potassium, magnesium, and calcium), lipid profile (LDL, HDL, and triglycerides), and CRP did not show significant changes at six months compared to baseline. In contrast, ESR decreased significantly, while ALP and GGT levels increased. Although AST and ALT remained stable, the rise in cholestatic enzymes necessitates clinical correlation and ongoing monitoring, particularly in geriatric patients with multimorbidity and polypharmacy.

Monitoring for viral hepatitis and tuberculosis is a critical aspect of safety assessment in geriatric patients undergoing biologic therapy, especially in the context of multimorbidity and age-related immune changes. Several real-world studies have reported variability in QFT results over time in patients with psoriasis receiving biologic therapy, including conversions during follow-up; however, the progression to active tuberculosis remains rare with appropriate screening and monitoring [15, 36–38]. Similar findings have been observed in geriatric cohorts, with baseline Interferon Gamma Release Assay (IGRA)/QFT positivity rates ranging from approximately 7% to 15%, and few or no cases of active tuberculosis reported in most studies [25, 39]. Additionally, a previous study indicated no cases of tuberculosis reactivation among patients treated with IL-17 or IL-23 inhibitors during follow-up [40]. In our geriatric cohort, QFT positivity was noted in a significant proportion of patients at baseline, with additional conversions from negative to positive observed during follow-up; however, no cases of active tuberculosis were detected during the study period. These findings suggest that changes in QFT status may occur during biologic therapy in geriatric patients and should be interpreted cautiously within the clinical context, supported by standardized screening and monitoring protocols. Notably, QFT conversion from negative to positive was observed in patients treated with biologic agents outside the anti-TNF-α group, indicating that careful monitoring for LTBI is warranted during biologic therapy in geriatric patients, regardless of biologic class.

The risk of HBV reactivation during biologic therapy is a significant safety concern in psoriasis, particularly among geriatric patients. Evidence suggests that HBV reactivation is relatively uncommon among anti-HBc–positive individuals receiving biologic agents, with systematic reviews indicating low rates of clinically significant reactivation [16, 41, 42]. However, data specifically addressing geriatric populations remain limited, and existing studies generally report a low incidence of overt hepatitis when appropriate screening and monitoring strategies are employed [25]. In our geriatric cohort, serologic changes related to HBV reactivation were observed during biologic therapy, highlighting the necessity of thorough baseline assessment and vigilant virologic monitoring in this demographic. Importantly, these serologic changes were not linked to clinically overt hepatitis or HBV-related adverse outcomes. These findings should be interpreted as indicators for treatment-related safety monitoring rather than direct measures of infection risk.

Among the four patients who experienced HBV reactivation, three were HBsAg-negative and anti-HBc-positive at baseline, receiving guselkumab, etanercept, or infliximab, while the fourth patient, who was HBsAg-positive, was treated with etanercept. Serologic changes occurred despite the administration of antiviral prophylaxis.

These observations imply that while antiviral prophylaxis diminishes the risk of HBV reactivation, it may not entirely eliminate it. Consequently, patients undergoing biologic therapy should be closely monitored for HBV reactivation, even when prophylaxis is in place. Furthermore, although anti-IL-23 agents are generally perceived to have a lower risk of reactivation compared to anti-TNF-α therapies, HBV reactivation can still occur in patients treated with these agents, necessitating consideration in clinical risk assessments and monitoring strategies. Since loss of anti-HBs may serve as an early serologic indicator of HBV reactivation, serial monitoring of anti-HBs titers is clinically significant. In settings where routine HBV DNA testing is not consistently accessible, tracking changes in anti-HBs levels may offer a practical adjunct for early detection and risk stratification of HBV reactivation.

This study has several limitations. Its retrospective, single-center design restricts causal inference and generalizability, and the six-month follow-up period limits conclusions regarding long-term efficacy and infectious outcomes. Patients were selected using a quota sampling approach to achieve balanced biologic groups, which may not accurately represent real-world treatment distributions. Subgroup and agent-level comparisons were further hampered by sample size, particularly given the relatively small number of patients within each biologic class; moreover, treatment allocation was not randomized, introducing potential confounding related to clinical decision-making. Adjustment for previous treatment exposure was constrained by a lack of detailed information on prior conventional systemic therapies prior to biologic initiation. Additionally, detailed adverse event data beyond treatment discontinuation and serious infection/malignancy outcomes were not systematically collected, which may limit the comprehensiveness of the safety assessment. The findings regarding blood count–derived inflammatory indices should also be interpreted cautiously due to the modest sample size and short follow-up duration. The limited number of patients with HBV reactivation prevents robust conclusions about class-specific reactivation risk. Nevertheless, this follow-up period enabled the evaluation of early clinical response, short-term changes in systemic inflammatory indices, and initial safety signals related to hepatitis B serologic dynamics and latent tuberculosis screening in a real-world geriatric population.

## Conclusion

In this real-world geriatric cohort, biologic therapy was associated with clinical improvement at six months, accompanied by reductions in systemic inflammatory indices derived from routine blood counts, with broadly comparable responses across biologic classes. Although variability in QFT and HBV serologic markers was noted during follow-up, no cases of active tuberculosis, clinically overt hepatitis, treatment discontinuation, serious infections, or malignancies occurred during the six-month period. These findings suggest that biologic therapies may be effective and appear to have an acceptable short-term safety profile in geriatric patients with psoriasis, while underscoring the importance of standardized baseline screening and close monitoring during treatment. Prospective, multicenter studies with extended follow-up are needed to further elucidate class-specific effects and long-term safety in geriatric patients.

## Data Availability

The datasets generated and/or analysed during the current study are available from the corresponding author on reasonable request.
